# Pearl Millet Aquaporin Gene *PgPIP2;6* Improves Abiotic Stress Tolerance in Transgenic Tobacco

**DOI:** 10.3389/fpls.2022.820996

**Published:** 2022-03-09

**Authors:** Palakolanu Sudhakar Reddy, Mahamaya G. Dhaware, Kaliamoorthy Sivasakthi, Kummari Divya, Marka Nagaraju, Katamreddy Sri Cindhuri, Polavarapu Bilhan Kavi Kishor, Pooja Bhatnagar-Mathur, Vincent Vadez, Kiran K. Sharma

**Affiliations:** ^1^International Crops Research Institute for the Semi-Arid Tropics (ICRISAT), Patancheru, India; ^2^Department of Biochemistry, ICMR – National Institute of Nutrition, Hyderabad, India; ^3^Department of Biotechnology, Vignan’s Foundation for Science, Technology & Research (Deemed to be University), Vadlamudi, India

**Keywords:** canopy temperature depression (CTD), exudation rate, *Pennisetum glaucum*, *PgPIP2;6*, progressive drought stress, transpiration rate, transpiration efficiency, vapor pressure deficit

## Abstract

Pearl millet [*Pennisetum glaucum* (L) R. Br.] is an important cereal crop of the semiarid tropics, which can withstand prolonged drought and heat stress. Considering an active involvement of the *aquaporin* (AQP) genes in water transport and desiccation tolerance besides several basic functions, their potential role in abiotic stress tolerance was systematically characterized and functionally validated. A total of 34 *AQP* genes from *P. glaucum* were identified and categorized into four subfamilies, viz., plasma membrane intrinsic proteins (PIPs), tonoplast intrinsic proteins (TIPs), nodulin-26-like intrinsic proteins (NIPs), and small basic intrinsic proteins (SIPs). Sequence analysis revealed that *PgAQPs* have conserved characters of *AQP* genes with a closer relationship to sorghum. The *PgAQPs* were expressed differentially under high vapor pressure deficit (VPD) and progressive drought stresses where the *PgPIP2;6* gene showed significant expression under high VPD and drought stress. Transgenic tobacco plants were developed by heterologous expression of the *PgPIP2;6* gene and functionally characterized under different abiotic stresses to further unravel their role. Transgenic tobacco plants in the T_2_ generations displayed restricted transpiration and low root exudation rates in low- and high-VPD conditions. Under progressive drought stress, wild-type (WT) plants showed a quick or faster decline of soil moisture than transgenics. While under heat stress, *PgPIP2;6* transgenics showed better adaptation to heat (40°C) with high canopy temperature depression (CTD) and low transpiration; under low-temperature stress, they displayed lower transpiration than their non-transgenic counterparts. Cumulatively, lower transpiration rate (Tr), low root exudation rate, declined transpiration, elevated CTD, and lower transpiration indicate that *PgPIP2;6* plays a role under abiotic stress tolerance. Since the *PgPIP2;6* transgenic plants exhibited better adaptation against major abiotic stresses such as drought, high VPD, heat, and cold stresses by virtue of enhanced transpiration efficiency, it has the potential to engineer abiotic stress tolerance for sustained growth and productivity of crops.

## Introduction

Pearl millet [*Pennisetum glaucum* (L) R. Br.], which is a diploid (2*n* = 14) cereal crop from the Poaceae family, is the sixth most important cereal crop providing food security to over 500 million people across Africa and Asia. It is known to be a versatile dryland cereal used as multi-purpose crop for food, feed, fuel, construction material, and forage, especially in the arid and semiarid regions (Nambiar et al., 2011; Vadez et al., 2012). Its germplasm displays a high degree of genetic diversity and variable tolerance to most of the abiotic stresses, thereby providing better grain yield in areas that are too harsh for profitable production of other cereals like rice, wheat, sorghum, and maize. Pearl millet possesses abiotic stress tolerance due to efficient water conservation mechanisms (Kholová et al., 2010a; Reddy et al., 2017, 2021), which is considered as an efficient way of enhancing crop yield under terminal drought stress (Kholová et al., 2010a; Zaman-Allah et al., 2011b; Belko et al., 2012). Enhanced water conservation traits/genes that relate to hydraulic processes in the plant would be highly desirable for better drought adaptation of pearl millet cultivars (Kholová et al., 2010a,b, 2012; Reddy et al., 2017, 2021; Tharanya et al., 2018). The rapid response of the transpiration rate (Tr) to increased vapor pressure deficit (VPD) suggests a possible role of hydraulic signals mediating the Tr response upon rising VPD. These hydraulic signals are regulated by aquaporins (AQPs), the specialized membrane transporters that play a pivotal role in conducting water at the cellular level (Reddy et al., 2017; Shekoofa and Sinclair, 2018; Tharanya et al., 2018).

Aquaporins (AQPs) are members of the Major Intrinsic Proteins (MIP) family, which is represented in all kingdoms from archaea, animals to plants (Gomes et al., 2009; Maurel et al., 2015; Deshmukh et al., 2016). They are 23–31 kDa proteins having several specific characteristic features, including transmembrane helices (TM) linked by five inter-helical loops (LA-LE) and conserved dual aspargine-proline-alanine (NPA) motifs (Maurel et al., 2008). A number of AQPs have been identified in diverse plant species, including 35 in *Arabidopsis* (Johanson et al., 2001), 43 in maize (Chaumont et al., 2001), 34 in rice (Sakurai et al., 2005), 58 in *Populus* (Gupta and Sankararamakrishnan, 2009), 72 in soybean (Zhang et al., 2013), 41 in *Sorghum* (Reddy et al., 2015a), 57 in *Brassica rapa* (Sonah et al., 2017), 28 in *Beta vulgaris* (Kong et al., 2017), 65 in wheat (Madrid-Espinoza et al., 2018), 41 in *Setaria italica* (Singh et al., 2019), 39 in cucumber (Zhu et al., 2019), 76 in tobacco (De Rosa et al., 2020), and 38 in pomegranate (Liu et al., 2021). Plant AQPs show a high degree of gene multiplicity and assembled as tetramers that are typically divided into seven subfamilies, including plasma membrane intrinsic proteins (PIPs), tonoplast intrinsic proteins (TIPs), nodulin-26-like intrinsic proteins (NIPs), and small basic intrinsic proteins (SIPs) that have been identified in both primitive and higher plants (Anderberg et al., 2012). Hybrid intrinsic proteins (Hips), GlpF-like intrinsic proteins (GIPs) (Danielson and Johanson, 2008), and X-intrinsic proteins (XIPs) have only been identified in mosses as well as several dicotyledonous plants (Bienert et al., 2011), including soybean (Zhang et al., 2013), but were not identified in monocots. These subclasses have been defined based on sequence similarity as well as intracellular localization (Maurel et al., 2015). AQPs received special attention due to their association with water deficit conditions and correlated with the abiotic stress tolerance mechanism (Sreedharan et al., 2013; Afzal et al., 2016). Although a few AQPs have been identified and functionally characterized from crop plants, except for some preliminary reports, there is no detailed investigation on the functional validation of the *AQP* gene family in millets, including pearl millet (Reddy et al., 2017; Grondin et al., 2020).

Studies on AQPs have been demonstrated in both model and crop plants under different abiotic stresses, where the PIP family has been shown to play a major role in abiotic stress tolerance by modulating the water transport pathway (Fetter et al., 2004; Yaneff et al., 2014). The overexpression of the *RsPIP2;1* gene has been shown to enhance drought and salt tolerance in transgenic *Eucalyptus* (Tsuchihira et al., 2010), while *TdPIP1;1* and *TdPIP2;1* from durum wheat and *HvPIP2;5* from barley have been shown to improve salinity and osmotic stress tolerance in tobacco and *Arabidopsis*, respectively (Ayadi et al., 2011; Alavilli et al., 2016). Upon overexpression of AQPs from tomato (*SlPIP2;1, SlPIP2;7, or SlPIP2;5*) and rice (*OsPIP1 and OsPIP2*) in *Arabidopsis*, higher hydraulic conductivity levels and survival rates were displayed under drought stress conditions (Mosa et al., 2012; Li et al., 2016). Specific AQP inhibitors, such as HgCl_2_, AgNO_3_, and H_2_O_2_, have rapidly reduced the root hydraulic conductivity in chickpea and pearl millet (Sivasakthi et al., 2017a,^2020^; Tharanya et al., 2018). Grondin et al. (2020) reported that pearl millet genotypes contrasting for water use efficiency differed in AQP gene expression. PgPIP1;3 and PgPIP1;4 were significantly more expressed in the low-water-use efficiency line than the high-water-use efficiency line. In addition, the low-water-use efficiency line has more reduction of root hydraulic conductivity by azide (AQP inhibitor) treatment than the high-water-use efficiency line. Our earlier report, PgPIP2;3, showed higher transcript abundance in a high transpiration rate (TR) than the low TR line under high-VPD conditions (Reddy et al., 2017).

The overexpression of the *StPIP1* gene was shown to ameliorate drought tolerance in potato where it improved water use efficiency, increased non-structural carbohydrates, thereby minimizing carbon starvation, and increasing biomass yield (Wang et al., 2017). The ectopic expression of *MaPIP1;1* and *MaPIP2;7* was shown to display multiple abiotic stress tolerance in transgenic banana (Xu et al., 2020, 2021), while transgenic rice overexpressing *OsPIP1;3* promoted plant growth and water uptake (Liu et al., 2019). More recently, *PePIP2;7* from bamboo has been demonstrated to confer abiotic stress tolerance in yeast and *Arabidopsis* (Sun et al., 2021).

Since pearl millet is a robust cereal crop of the semiarid tropics, it is important to understand different components of the abiotic stress tolerance mechanism. We systematically analyzed the sequences and relative expression of all *PgAQPs* under high VPD and progressive drought stress, besides the functional characterization of the *PgPIP2;6* gene in tobacco. This work provides insights on the molecular basis of water conservation mechanisms in pearl millet.

## Materials and Methods

### Identification of Pearl Millet Aquaporin Genes

Pearl millet *AQP* gene sequences were retrieved using maize, rice, and sorghum *AQP* genes as a search query and scanned against the pearl millet genome by NCBI blast N, X, and P databases. Chromosomal locations of PgAQPs were determined with the information obtained from the pearl millet genome database^1^. The intron-exon structures were predicted using the Gene Structure Display Server^2^ (Hu et al., 2015), and the MOTIF search^3^ was used to retrieve the conserved domains. Sequence analysis, including the open reading frame (ORF) and amino acid (AA) translations, molecular weight, and isoelectric point (pI) of *PgAQP* genes, was analyzed using the MacVector software (V16.09). The GRAVY (grand average of hydropathicity) instability and aliphatic indices were predicted by employing ProtParam of Expasy tools^4^ (Gasteiger et al., 2005). WoLFPSORT II^5^ was used to predict the subcellular locations (Guo et al., 2015), while the TMHMM server^6^ was used to screen the putative *trans*-membrane helices (Möller et al., 2001). Phosphorylation sites (serine, threonine, and tyrosine) of the PgAQPs were searched using the NetPhos 3.1^7^ program (Bloom et al., 2004). Multiple sequence alignment (MSA) and phylogenetic analysis were carried out with full-length protein sequences of closely related AQPs from monocots and dicots using the ClustalW program of MacVector and MEGA 6.0 (Tamura et al., 2013). Bootstrap analysis was performed using 1,000 replicates, and the branch lengths corresponded to phylogenetic distances in units of the number of amino acid substitutions per site. Conserved motifs were identified with the help of the MEME suit^8^ with parameters such as the number of motifs 10, width of the motifs set in-between 3 and 60, while parameters were set as default (Bailey et al., 2009). PAL2NAL software was used to calculate the synonymous and non-synonymous sites, their substitution rates, and dN/dS for the PgAQPs orthologs and paralogs (Suyama et al., 2006). Promoter regions of all the identified *PgAQP* genes were searched by PlantCARE^9^ (Lescot et al., 2002). The 3D structure of PgPIP2;6 protein was modeled using SWISS-MODEL^10^, viewed and labeled using the PyMol viewer, and ligands of the protein were searched using SWISS-MODEL software. Online software tools, such as PatchDock^11^ and FireDock^12^, were used to dock the model against its ligand. For converting the Structure Data File (SDF) into Protein Data Bank (PDB) format, Marveanbeans software was used, while for docking the model with ligand PatchDock, an online server was utilized and the results were visualized by PyMol.

### Plant Material and Abiotic Stress Treatments

In a previous study, we have selected two pearl millet genotypes (ICMR 1152 and ICMR 1122), contrasting for VPD (Serraj et al., 2005; Reddy et al., 2017, 2021). These genotypes were used to express *PgAQPs* under high VPD and progressive drought stress. In the case of tobacco, both the transgenic and wild-type plants were grown in controlled glasshouse conditions at 26°C (14-h day) and 20°C (10-h night) 45 DAS. These plants were further subjected to progressive drought stress (Kholová et al., 2010a) and high VPD (Reddy et al., 2017, 2021) where the controls were maintained at 26°C for high-VPD treatment. Leaf and root tissues from the respective plants were collected and immediately snap frozen in liquid nitrogen for total RNA extraction and qRT-PCR analysis. Three biological replicates were maintained and collected for each stress treatment.

### Cloning and Genetic Transformation of *PgPIP2;6* Gene

Based on the expression profile, *PgPIP2;6* gene was selected and PCR amplified from the pearl millet cDNA and cloned between the CaMV35S - Nos terminator in the Gateway-compatible entry vector (pL12R34H-Ap) by digesting with *Nde*I and *Not*I restriction enzymes. The expression cassette of *PgPIP2;6* gene (CaMV35S:*PgPIP2;6*:NosT) from the entry vector was further cloned into the Gateway-based plant transformation vector pMDC100 using LR Gateway cloning. The recombinant plasmid was transformed into *Agrobacterium tumefaciens* strain EHA105 by electroporation and used for tobacco transformation as reported previously (Divya et al., 2019); the selected rooted shoots were transferred to pots containing autoclaved sand and soil (1:1) mixture and maintained in a containment greenhouse until flowering and the seed set.

### Growth of Tobacco Transgenic Plants

The untransformed wild type (WT) plants were used as negative control and a recombinant plasmid containing the *PgPIP2;6* expression cassette as positive control. Fully grown transgenic tobacco plants (T_0_ generation) were self-pollinated using paper bags to produce T_1_ seeds. Progeny plants (T_1_) selected with kanamycin (50 mg L^–1^) on an agar medium were grown in a growth chamber at 25°C at a photoperiod of a 16-h light and 8-h dark cycle, followed by their advancement to T_2_ generation for further analysis. The T_2_ generation plants and their untransformed wild types were maintained at 25–27°C with a 16-h light and 8-h dark cycle with 300 μmol m^–2^ s^–1^ light and 70-80% relative humidity (RH) in a biosafe glasshouse and used for physiological and molecular analysis. The genomic DNA of the putative T_0_ tobacco transgenic plants (20 days) was isolated using the DNeasy Plant Mini Kit (Qiagen, Germany). Primer sets for *CaMV35S_F* (GATATCGTACCCCTACTCCAA)- *PgPIP2;6_R* (AGAAGACGGTGTAGACGAGCA) and *NptII_F - NptII_R* were used to confirm the transgene integration.

### Measurement of Vapor Pressure Deficient

The T_2_ generation transgenic tobacco plants and the WT plants were grown in 8-inch plastic pots filled with 5.5 kg of Alfisol where 16 replicate pots of each genotype were grown and the 10 most uniform plants were selected to design the experiments in controlled environment growth chambers. One day before the experiment, the pots were well watered, allowed to drain to reach a field capacity, and bagged with a transparent plastic bag wrapped around the plant stem to prevent the evaporation of soil water during the evaluation of plant transpiration. In controlled growth chambers, the transpiration rate response to stable and increasing VPD is carried out; for stable VPD, the chamber was maintained at 1.2 kpa throughout the experiment, whereas, for increasing VPD or treatment, the chamber VPD was raised from 0.6 to 3.80 kPa gradually (Supplementary Table 5). Ten replications of wild-type and transgenic tobacco plants were used in both stable and increasing VPD chambers to measure the plant transpiration response throughout the day. An equal number of WT plants were also maintained in two chambers where one chamber was maintained at 1.2 kPa throughout the experiment (a low-VPD chamber), while the other had a ladder of VPD ranging from 0.69 to 3.8 kPa (a high-VPD chamber) with the VPD stress imposed as described earlier (Reddy et al., 2017, 2021) (Supplementary Table 5). Within each VPD level, the plants were weighed gravimetrically every 60 min with 0.01-g precision scales (KERN 3600-2N, Kern & Sohn GmbH, Balingen, Germany) to derive transpiration values from consecutive weighing. Between successive VPD levels, a 15-min transition was allowed to gradually increase the VPD to the next level. A data logger (Lascar Electronics Inc., United Kingdom) was positioned within the plant canopies in the growth chamber for regular records of air temperature and relative humidity throughout the measurement period. At the end of the VPD ladder (3.8 kPa at 03:00 pm), root and leaf samples were collected from low- (1.2 kPa) and high-VPD chambers (3.8 kPa), frozen immediately in liquid nitrogen, and stored at −80°C for RNA isolation. The plants were harvested at the end of transpiration measurement. The leaf area was measured by a leaf area meter (LI-3100C area meter, LI-COR^®^Biosciences, United States). At the end of the VPD experiment, shoots from low- and high-VPD chambers were cut using a razor blade. The root exudate (xylem sap) was collected for 45 min using pre-weighed Eppendorf^®^ cones stuffed with tissue paper (Kimtech Science, Ontario, United States). The root exudation rate was calculated by normalizing the amount of sap exuded by the root surface area and time. The root surface area (cm^2^) and root volume (cm^3^) were calculated by scanning the roots with Shimadzu scanner and analyzing with Winrhizo software (Winrhizo, Regent Ltd., Canada).

### Imposition of Abiotic Stresses

#### Progressive Drought Stress

Progressive drought stress was imposed on 45-day-old tobacco plants according to Kholová et al. (2010a). From the nine well-watered (WW) replicate pots, six were used for physiological measurements and three for qRT-PCR analysis. The plants from WW pots were maintained by daily re-watering up to an 80% field capacity by bringing pot weight to 100 g below the field capacity weight. For water stress (WS) treatment, 12 pots were exposed to the soil moisture stress, from which nine were kept for physiological measurements and three used for collecting tissue samples for gene expression analysis. For WS, the plants were partially compensated for transpiration, i.e., the plants were allowed to lose no more than 50-g water each day, and any transpiration loss of over 50 g was added back to the pots as described earlier (Vadez and Sinclair, 2001) to allow for progressive development of water-deficit over a 2-week period to measure parameters like normalized transpiration ratio (NTR) and fraction of transpirable soil water (FTSW) (Kholová et al., 2010a). When the NTR value of the test plants neared 0.1, which was an indication of WS (pots reached 10% water content), leaves, as well as root tissues, were collected and snap frozen in liquid nitrogen and stored at −80°C until RNA extraction.

#### Heat Stress

The plants grown in a glasshouse for 45 days were exposed to heat stress at 40°C for 4 h and RH of 35%. Other set of plants was kept in control conditions in the growth chamber (28°C and 70% RH). Transpiration in both heat-stressed and control-chamber-grown plants was measured by weighing the pots before and after heat stress treatments. In addition, thermal images (infra-red) were taken in both heat-stressed and control plants in the chamber using infrared (IR) FlexCam S (Infrared Solutions, Plymouth, MN, United States) with a sensitivity of 0.09°C with an accuracy of ± 2%. SmartView 2.1.0.10 software (Fluke Thermography Everett, WA, United States) was used for the analysis of thermal images and estimation of canopy temperatures (Zaman-Allah et al., 2011a; Sivasakthi et al., 2017b). Leaf and root tissues from the control and heat stressed plants were collected, snap frozen in liquid nitrogen, and stored at −80°C for RNA extraction.

#### Cold Stress

One set of 45-day-old plants was exposed to 4°C for 4 h in the cold room, while the other set of plants was maintained in a growth chamber (28°C and 70% RH) as controls. Transpiration in both cold-stressed and control plants was measured before and after stress treatment. Leaf and root tissues from the control and cold-stressed plants were collected, snap frozen in liquid nitrogen, and preserved at −80°C for RNA extraction.

### Quantitative Real-Time PCR Analysis

Total RNA was isolated from pearl millet plants (VPD sensitive and insensitive genotypes treated with high VPD and progressive drought stress) and transgenic tobacco plants (control and treated) using 100 mg of tissue with RNeasy Plant Mini kit (Qiagen, Germany). The quantity and the quality of RNA were analyzed using a NanoVue plus spectrophotometer (GE Health Care, United States) and the ratio of the absorbance measured at 260 and 280 nm (260/280) of the samples ranging from 1.8 to 2. The integrity of the RNA was further verified through denaturing 1.4% agarose gel and Bioanalyzer. The total RNA isolated from control and treated samples was diluted to 30 ng/μl concentrations for the direct use in qRT-PCR reactions performed in the optical 96-well plates on a Realplex (Eppendorf, Germany). Reactions were performed in a total volume of 10 μl, containing 1 μl of RNA (30 ng), 400 nM of each primer, 5 μl of 2 × one step SYBR RT-PCR buffer 4 (Takara, Japan), and 0.5 μl of Prime Script One Step Enzyme Mix 2 (Takara, Japan), and final volume made up to 10 μl with RNase-free H_2_O. qRT-PCR reactions of all the samples were carried out by following a standard thermal profile: 42°C for 5 min and 95°C for 10 s (reverse transcription), followed by 40 cycles of 15 s at 95°C, 15 s at 62°C with a fluorescent signal recording and 15 s at 72°C. Amplicon dissociation curves were recorded after 40 cycles by heating from 58 to 95°C with fluorescence measured within 20 min. The experiments were independently repeated three times, and the data from these experiments were averaged. The relative expression levels of *PgAQP* genes in response to high VPD and progressive drought stress in Tr contrasting genotypes were estimated using qBase software (Hellemans et al., 2007) by normalizing with corresponding control samples as well as with reference genes (Reddy et al., 2015b). Relative expression levels of the *PgPIP2;6* gene in response to different tissues of transgenic tobacco and abiotic stress treatments were estimated using the 2^–ΔΔCt^ method (Livak and Schmittgen, 2001) by normalizing with *L25* and *EF-1 alpha* reference genes (Schmidt and Delaney, 2010).

### Statistical Analysis

Transpiration response to increasing VPD (a high-VPD chamber) was analyzed with non-linear regression of Graph pad Prism version 6 (Graph Pad software, Inc., CA, United States), which provides *r*^2^, a break point, and slope values. For the progressive drought stress experiment, the relationship between NTR and FTSW was analyzed with non-linear regression of Graph pad Prism version 6 (Graph pad software, Inc., CA, United States), which provides breakpoint values (Kholová et al., 2010b). Software SmartView 2.1.0.10 (Fluke Thermography) was used for analysis of the thermal images and the estimation of canopy temperatures (Zaman-Allah et al., 2011a). CTD was calculated as the difference between the air temperature (T) and leaf temperature (CTD = T air – T leaf). The relative expression of the *PgPIP2;6* gene under different abiotic stresses was analyzed according to Livak and Schmittgen (2001). All the data from stress experiments (VPD, drought, heat, cold) and relative expressions were analyzed with statistical program package CoStat version 6.204 (Cohort Software, Monterey, CA, United States). One-way ANOVA was carried out to test for genotypic difference among the transgenic events and WT. The means were compared using Tukey-Kramer test and least significant difference (LSD) (at *p* ≤ 0.05).

## Results

### Identification of *Aquaporin* Gene Family and Sequence Analysis

**FIGURE 1:**
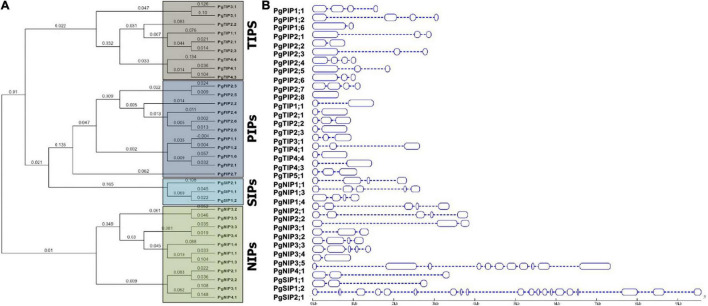
Characterization of pearl millet AQP proteins. **(A)** The phylogenetic tree was constructed using MacVector software by the NJ method. **(B)** Exon and intron structure of pearl millet *AQP* genes is indicated by blue rectangles and thin dotted lines, respectively. The scale bar indicates nucleotide size length in the base pairs.

A phylogeny of AQPs was carried out to understand the evolutionary relationships among pearl millet AQPs within *Pennisetum* and with that of sorghum, maize, and rice (Figure 1A and Supplementary Figure 1). PgAQP’s were grouped into four major subfamilies based on their conserved and high similarity index, including PgPIPs, PgTIPs, PgNIPs, and PgSIPs (Figure 1A). A total of 34 *PgAQP* genes were identified in pearl millet and named based on their relationship with their counterparts in the related species (Table 1 and Supplementary Figure 1). Among all classes, the PgPIP subfamily occupied the highest number of AQP genes with 11 members. Exon/intron structure displayed a range of 1–19 (PgSIP2;1) introns, the highest number present in the PgNIP subfamily and the lowest in the PgTIP subfamily (Table 1 and Figure 1B). The identified AQPs were distributed on six chromosomes of pearl millet. Of the 34, 3 were located on Chromosome 1,7 on 2,15 on 3,5 on 4, 2 on 5, and 1 on 6. While Chromosome 3 was the hot spot with a maximum number of *AQP* genes with most PIPs and TIPs, Chromosome 7 did not show any *AQP* genes (Table 1). PgAQPs ORFs ranged from 636 bp (PgPIP2;8) to 2,823 bp (PgNIP4;1) with an average size of 528 bp. PgAQP proteins consisted of amino acids, ranging from 211 (PgPIP2;8) to 940 (PgNIP4;1), majority of them being basic, where the pI values varied between 4.9 (PgNIP1;4) and 9.7 (PgPIP2;2). The MWs ranged from 21K Da to 99.79K Da with an instability index varying from 20.68 (PgTIP2;2) to 41.75 (PgNIP4;1), which was the only unstable one. The GRAVY values indicated that most of them were hydrophobic except PgSIP2;1, the only hydrophilic AQP, and all exhibiting the highest aliphatic index (Table 1). Most of the PgAQPs showed the presence of serine, threonine, and tyrosine, where serine was the most dominating phosphorylation site (Supplementary Table 1). The highest number of serine residues was observed in the PgNIP subfamily, followed by PIPs and TIPs. Protein kinase C (PKC) and an unspecific kinase were the major types present in all the proteins. The common kinases in all subfamilies were next to PKC, cdc2, PKA, CDK1, DNAPK, P38MAPK, and PKG (Supplementary Table 1). Localization of PgAQPs indicated that, while they were abundant in the plasma membranes with an average of six transmembrane helices, only NIP3 subfamily members were found in the vacuole (Table 1). Motif organization of the four subfamilies of PgAQP was deduced by submitting protein sequences to MEME^13^. A total of 10 conserved motif sequences were identified in 34 PgAQP proteins, showing group-specific distribution. Motifs 1 and 5 were the most conserved across all PgAQPs subfamilies positioned at C-terminus (Supplementary Figure 2). While motifs 6 and 7 were conserved in all the PgTIPs and PgNIPs, motifs 2 and 8 were noticed at the PgPIP subfamily’s N-terminus and Motif 9 in TIP and NIP subfamilies. Motif 3 was common in all the groups situated in the middle (Supplementary Figures 3, 4). All PgAQP proteins had the inherent conserved NPA motif (Motifs 1 and 3) and the aromatic/arginine (ar/R) selectivity filter (Supplementary Figures 2, 3).

**TABLE 1:** Physico-chemical characteristics of PgAQPs.

S. no	Accession number	Gene	Subfamily	ORF (bp)	No. of Introns	Chromosome	Chromosome localization	Protein (AA)	DBD	MWt (kDa)	pI	GRAVY	Aliphatic index	Instability index	TM helices	Localization
1	Pgl_GLEAN_10010809	*PgPIP1;1*	PIP1	867	3	3	5721622–5724718	288	46–215	30.76	8.82	0.349	94.24	34.84	6	Plas
2	Pgl_GLEAN_10001520	*PgPIP1;2*	PIP1	867	3	3	274573998–274575598	288	46–215	30.7	9.13	0.364	96.28	29.35	6	Plas
3	Pgl_GLEAN_10005724	*PgPIP1;6*	PIP1	909	1	2	104247932–104248932	302	54–287	31.31	7.72	0.425	95.03	28.06	6	Plas
4	Pgl_GLEAN_10028064	*PgPIP2;1*	PIP2	873	2	3	12453415–12456334	290	35–271	30.35	7.69	0.551	103.03	27.03	6	Plas
5	Pgl_GLEAN_10009812	*PgPIP2;2*	PIP2	699	1	2	64966663–64967453	232	24–208	24.59	9.66	0.445	102.59	25.79	5	Vacu
6	Pgl_GLEAN_10028876	*PgPIP2;3*	PIP2	867	2	3	45603209–45606038	288	33–270	30.11	6.95	0.523	103.12	28	6	Plas
7	Pgl_GLEAN_10028056	*PgPIP2;4*	PIP2	765	3	3	12167791–12168852	254	32–236	26.6	6.95	0.499	101.06	24.47	5	Plas
8	Pgl_GLEAN_10035675	*PgPIP2;5*	PIP2	873	2	3	257669631–257671533	290	34–271	30.38	6.58	0.502	103.72	27.26	6	Plas
9	Pgl_GLEAN_10028055	*PgPIP2;6*	PIP2	861	2	3	12156953–12158005	286	32–268	29.93	8.31	0.555	101.71	28.32	7	Plas
10	Pgl_GLEAN_10010255	*PgPIP2;7*	PIP2	861	3	Scaffold 763	240584–241753	286	36–264	29.79	9.16	0.668	107.76	28.95	6	Plas
11	Pgl_GLEAN_10028060	*PgPIP2;8*	PIP2	636	0	3	12322656–12323291	211	32–203	21.79	5.05	0.588	105.45	24.52	4	Plas
12	Pgl_GLEAN_10002147	*PgTIP1;1*	TIP1	750	1	5	153303973–153305471	249	17–232	25.72	6.27	0.812	107.75	25.92	6	Plas
13	Pgl_GLEAN_10009584	*PgTIP2;1*	TIP2	750	1	3	33622445–33623295	249	14–231	25.05	6	0.918	114.9	20.82	6	Plas
14	Pgl_GLEAN_10030617	*PgTIP2;2*	TIP2	747	2	3	100540239–100541171	248	15–231	25.02	5.66	0.924	108.95	20.68	6	Plas
15	Pgl_GLEAN_10000631	*PgTIP2;3*	TIP2	744	1	2	30657555–30658398	247	14–231	24.87	6.16	0.915	110.69	24.87	6	Plas
16	Pgl_GLEAN_10028702	*PgTIP3;1*	TIP3	675	2	2	44624536–44625481	224	20–196	23.15	6.39	0.49	98.17	34.15	5	Plas
17	Pgl_GLEAN_10002901	*PgTIP4;1*	TIP4	681	2	1	263875434–263878070	226	23–208	23.95	7.8	0.631	106.95	21.78	5	Plas
18	Pgl_GLEAN_10003218	*PgTIP4;4*	TIP4	750	1	3	148807632–148808412	248	16–231	25.1	6.74	0.825	114.92	22.65	6	Vacu
19	Pgl_GLEAN_10003219	*PgTIP4;3*	TIP4	744	1	3	148813464 –148814777	247	12–223	25.14	7.13	0.856	116.52	30.23	6	Plas-Vacu
20	Pgl_GLEAN_10033583	*PgTIP5;1*	TIP5	813	2	3	272599938–272601028	270	14–243	26.68	8.91	0.808	101.04	29.29	6	Plas
21	Pgl_GLEAN_10012175	*PgNIP1;1*	NIP1	846	3	2	195368171–195370486	281	37–248	29.51	9.1	0.401	94.48	30.87	6	Plas
22	Pgl_GLEAN_10028618	*PgNIP1;3*	NIP1	822	4	1	261387113–261389753	273	44–247	28.76	8.35	0.562	100.81	29.52	6	Plas
23	Pgl_GLEAN_10028339	*PgNIP1;4*	NIP1	792	3	3	145385690–145386826	263	56–242	27.93	4.86	0.5	96.84	34.9	5	Plas
24	Pgl_GLEAN_10018521	*PgNIP2;1*	NIP2	891	4	3	14105646–14109009	296	46–253	31.81	6.74	0.39	97.53	37.58	6	Plas–ER
25	Pgl_GLEAN_10019286	*PgNIP2;2*	NIP2	894	4	2	103033018–103036838	297	53–258	31.56	7.69	0.452	93.27	37.87	6	Plas
26	Pgl_GLEAN_10034621	*PgNIP3;1*	NIP3	909	2	2	40497062–40500916	302	70–281	31.42	9.1	0.433	96.32	34.68	5	Plas
27	Pgl_GLEAN_10030881	*PgNIP3;2*	NIP3	864	2	4	55573901–55575272	287	72–283	29.29	6.97	0.687	106.52	29.84	6	Vacu
28	Pgl_GLEAN_10030882	*PgNIP3;3*	NIP3	942	3	4	55609410–55610656	313	74–286	32.83	8.5	0.684	118.79	27.27	6	Vacu
29	Pgl_GLEAN_10030883	*PgNIP3;4*	NIP3	933	4	4	55648949–55650374	310	81–282	32.99	8.41	0.497	106.68	31.56	5	Vacu
30	Pgl_GLEAN_10030872	*PgNIP3;5*	NIP3	837	1	4	55508562 –55509419	278	64–274	27.79	8.02	0.664	105.86	36.79	6	Vacu
31	Pgl_GLEAN_10012100	*PgNIP4;1*	NIP4	2823	10	6	110090448 –110091766	940	438–641	99.79	7.79	0.172	103.13	41.65	6	Plas
32	Pgl_GLEAN_10014008	*PgSIP1;1*	SIP1	717	2	4	93394125–93397493	238	53–234	24.45	8.43	0.702	107.18	27.71	6	Plas
33	Pgl_GLEAN_10003744	*PgSIP1;2*	SIP1	726	2	1	175152169–175154986	241	4–225	25.32	9.54	0.808	113.44	30.32	6	Plas
34	Pgl_GLEAN_10026167	*PgSIP2;1*	SIP2	2604	19	5	126489209 126491163	867	692–859	94.65	5.63	−0.049	84.07	36.44	5	ER

*ORFs, number of amino acids (bp), proteins (amino acids), chromosomal location, isoelectric point (pI), molecular weight (MW), DNA binding domains (DBD), number of introns, localization, GRAVY, instability index, aliphatic index, and transmembrane helices.*

A total of 13 paralogs were identified where five were from NIPs, four from PIPs, three from TIPs, and one from SIP. The paralog’s duplication events were grouped into regional and segmental gene duplications based on their chromosomal localization and phylogenetic analysis. Of the 13 events, five were regional duplications; PgPIP1;1/PgPIP1;2, PIP2;3/PIP2;5, and PIP2;6/PIP2;8 were noticed on Chromosome 3 and NIP3;2/NIP3;5, and NIP3;3/NIP3;4 on Chromosome 4. The remaining eight were segmental duplication events (Supplementary Table 1). Furthermore, among the non-synonymous to synonymous substitutions, out of 13, 9 events exhibited purifying/stabilizing selection (<1), while the remaining four were positive/Darwinian selection (>1). The evolutionary relationship of the pearl millet with others showed a class-specific, group-specific relationship (Supplementary Table 1). Of the 12 orthologous events, six had an orthologous relationship with sorghum while three each with maize and rice. There were only three orthologous duplication events (PgPIP2:7/SbPIP2:7; PgTIP5:1/SbTIP5:1, and PgNIP3:2/SbNIP3:2) that showed positive selection, while the remaining nine events exhibited Darwinian selection pressure, purifying/stabilizing selection during evolution. While sorghum and maize showed 16 orthologous events, sorghum and rice exhibited four events, rice and maize exhibited one orthologous event (Supplementary Figure 1).

### Promoter Analysis

To understand the regulatory mechanism of *PgAQP* genes of pearl millet, *cis*-acting elements specific to tissue and abiotic stress within the upstream regions of *PgAQP* genes were identified (Supplementary Table 4). Up to seven Skn1 elements specific to endosperm expression were detected in majority of the *PgAQP* genes. GCN4 was another endosperm specific element present only in the *PgPIP* genes (*PgPIP2;1*, *PgPIP2;2*, *PgPIP1;1*, and *PgPIP1;2*). CAT-box and CCGTCC-box related to meristem expression were noticed in the promoter regions of all the *PgAQP* genes. Along with tissue-specific *cis*-elements, several abiotic stress-inducible *cis*-elements were detected in the promoter regions of the *PgAQP* genes. HSE *cis*-element involved in heat stress was observed in the promoter regions of 6 *PgAQP* genes (*PgPIP1;1*, *PgPIP2;1*, *PgTIP1;1*, *PgTIP2;2*, *PgNIP1;3*, and *PgNIP3;4*). The cold-responsive LTR element was noticed in 11 *PgAQP* genes (*PgPIP1;1*, *PgPIP1;6*, *PgPIP2;4*, *PgPIP2;7*, *PgPIP2;8*, *PgTIP2;1*, *PgTIP2;2*, *PgTIP2;3*, *PgTIP4;1*, *PgNIP3;1*, and *PgSIP1;1*). While over 50% of the *PgAQP* genes had drought-inducible MBS elements, the ABRE *cis*-acting element involved in the abscisic acid (ABA)-inducibility was detected in the promoter regions of 15 *PgAQP* genes. The TCA *cis*-element involved in salicylic acid responsiveness was noticed in the promoter regions of the four *PgAQP* genes. CGTCA and TGACG-motifs involved in the MeJA responsiveness were recognized in most of the upstream regions of *PgAQP* genes. Several core promoter regions were observed in *PgAQP* promoters, including TATA-box, CCAAT-motif, and TC-rich repeats (Supplementary Table 4).

### Expression of *PgAQP* Genes Under Progressive Drought and High Vapor Pressure Deficit Stress in Pearl Millet

#### Progressive Drought Stress

**FIGURE 2:**
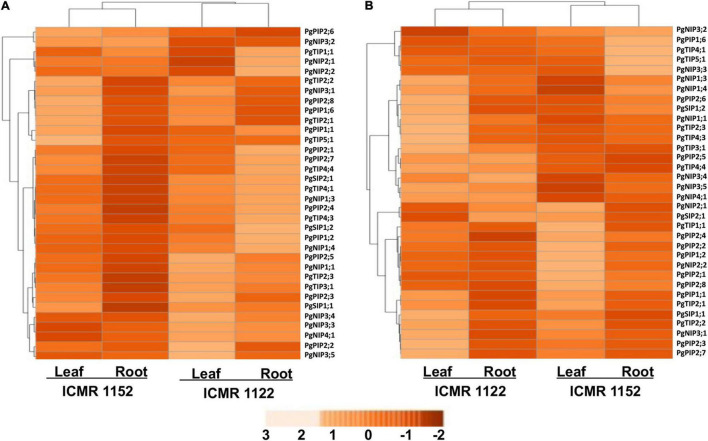
An expression profile of the 34 *PgAQP* genes in leaves and roots of VPD-sensitive and VPD-insensitive genotypes under **(A)** drought stress and **(B)** high VPD. The color bar represents normalized log2 values.

The expression profiling of *PgAQPs* was investigated among VPD-insensitive - ICMR 1122 and VPD-sensitive - ICMR 1152 genotypes in leaf and root tissues under progressive drought and high-VPD stresses using qRT-PCR. To understand the function of *AQP* genes in drought stress adaptation/tolerance of pearl millet, progressive drought stress was imposed in pearl millet genotypes (ICMR 1122 and ICMR 1152), contrasting for transpiration rate response to increasing VPD. Many *PgAQP* genes showed upregulation in both leaf and root tissues upon drought imposition (Figure 2A). *PgPIP2;6* of ICMR 1152 and *NIP1;3* and *PgNIP1;3* of ICMR 1122 exhibited the highest level of expression in leaf as well as root tissues. Similarly, drought stress caused upregulation of six *PgAQP* genes like *PgPIP2;5*, *PgPIP2;6*, *PgNIP3;2*, *PgNIP3;3*, *PgNIP3;5*, *PgNIP4;1*, in both the tissues of the genotype ICMR 1152. *PgTIP2;3*, *PgPIP2;8*, and the *PgTIPs*, such as *PgNIP1;1*, *PgNIP1;3*, *PgNIP3;1, PgNIP3;2*, and *PgSIP1;1*, were upregulated only in leaf tissues. Notably, *PgPIP1;2*, *PgPIP2;5*, *PgPIP2;6*, *PgTIP2;3*, *PgTIP3;1*, *PgTIP4;1*, *PgTIP4;3*, *PgTIP4;4*, *PgNIP1;1*, *PgNIP1;4*, *PgNIP1;3*, *PgNIP2;2*, *PgNIP3;3*, *PgNIP3;4*, *PgNIP3;5*, *PgNIP4;1*, *PgSIP1;1, PgSIP1;2*, and *PgSIP2;1* exhibited higher expressions in root tissues of the genotype ICMR 1122. In contrast, only one gene, *PgNIP3;1*, showed high transcript levels in the leaf tissues of same genotype. All genes recorded higher expressions in the ICMR 1152 genotype compared to ICMR 1122. The least expression/downregulation was noticed in almost all AQPs except a few in the leaf tissues of the ICMR 1122 genotype. In summary, differential expression patterns were observed in root tissues of ICMR 1122 and leaf and root tissues of ICMR 1152 (Figure 2A).

#### High Vapor Pressure Deficit

Under high-VPD conditions, *PgAQP* genes showed differential expressions where the *AQP* genes, viz., *PgPIP1;6, PgPIP2;6, PgTIP4;1, PgTIP5;1, PgNIP1;1, PgNIP1;3, PgNIP1;4, PgNIP3;2*, and *PgNIP3;3*, were upregulated in the root tissues of ICMR 1152 where expressions were highest among all the 34 tested *PgAQPs.* Interestingly, *PgTIP3;1*, *PgTIP4;4*, and *PgNIP3;4* showed moderate or high expressions in roots and leaf tissues of ICMR 112 in comparison with ICMR 1152 (Figure 2B). Apart from these, 10 genes, namely, *PgPIP1;1, PgPIP1;2, PgPIP2;2, PgPIP2;7, PgPIP2;8, PgTIP1;1, PgTIP2;1, PgTIP2;2, PgTIP4;1*, and *PgNIP3;2*, displayed higher expression levels in the leaf tissues of ICMR 1152. Among them, *PgPIP1;1; PgTIP1;1, PgTIP2;1, PgTIP2;2* and *PgNIP3;2* exhibited expressions in the leaf tissues of ICMR 1122. Genes, such as *PgNIP1;1*, *PgNIP1;3*, *PgNIP1;4*, *PgNIP3;2*, *PgNIP3;3*, *PgNIP3;4*, and *PgNIP5;1* exhibited significant upregulation in root tissues of both ICMR 1152 and ICMR 1122. *PgTIP4;1*, *PgNIP1;3*, and *PgNIP3;3* in ICMR 1152 and *PgTIP3;1*, *PgTIP4;4*, *PgNIP1;1*, *PgNIP1;3*, and *PgNIP4;1* in ICMR 1122 exhibited significant expressions in root tissues (Figure 2B). Other *PgAQP* genes revealed the lowest expression in both the tissues of ICMR 1122 upon high-VPD stress treatment. In general, the *PgAQP* genes were highly induced in root tissues than leaf tissues of both the genotypes in response to high-VPD stress.

### Cloning and Transformation of Tobacco With *PgPIP2;6*

**FIGURE 3:**
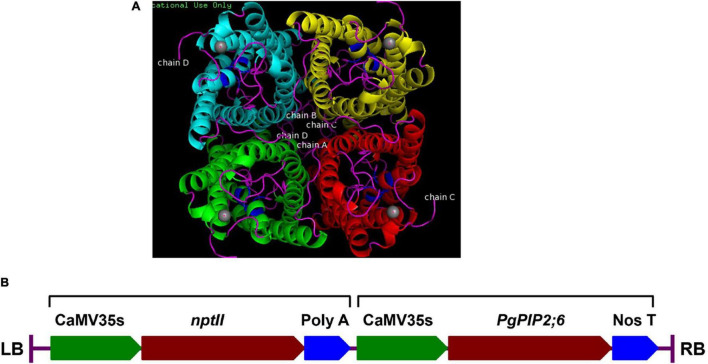
Characterization of the pearl millet *PgPIP2;6* gene**. (A)** The 3D model of *PgPIP2;6* protein built by using homology based *in silico* modeling. Aquaporin *PgPIP2;6* is incorporated into the membranes in a tetrameric arrangement, comprising four individual pores. The protein structure has classical aquaporin structure with conserved NPA motif regions, which are highlighted in blue. The ligand of the protein binds to the protein near the NPA motifs. Ligand (Cadmium ion) is highlighted in gray spheres. **(B)** Schematic representation of the T-DNA region of the plant transformation vector (pMDC100), showing the two expression cassettes for *nptII* and *PgPIP2;6* genes. RB, right border; 35SP, CaMV35s promoter; *PgPIP2;6*, *pearl millet plasma intrinsic protein 2;6* gene; *nptII*, *neomycin phosphotransferase* gene; NosT, Nos terminator; LB, left border.

Based on the gene expression profile, the gene *PgPIP2;6* was cloned into the pMDC100 vector under the control of *CaMV35S* promoter using the Gateway-cloning system. Plant expression cassette harboring CaMV35sP-*PgPIP2;6*-NosT (Figure 3) was transformed into tobacco and the putative transgenic plants confirmed by PCR with CaMV35S trans_F - *PgPIP2;6* trans_R and nptII F- nptII R primers for integration of the transgenes. Out of the 41 T_0_ transgenic plants, nine events in T_1_ were PCR positive that were analyzed through Mendelian segregation, and three T_2_ transgenic events (E 1–20, E 11–10, and E 28–9) were selected for further analysis.

### Physiological Performance of the *PgPIP2;6* Transgenic Tobacco Plants

#### Transpiration Response to Increasing and Stable Vapor Pressure Deficit Conditions

**FIGURE 4:**
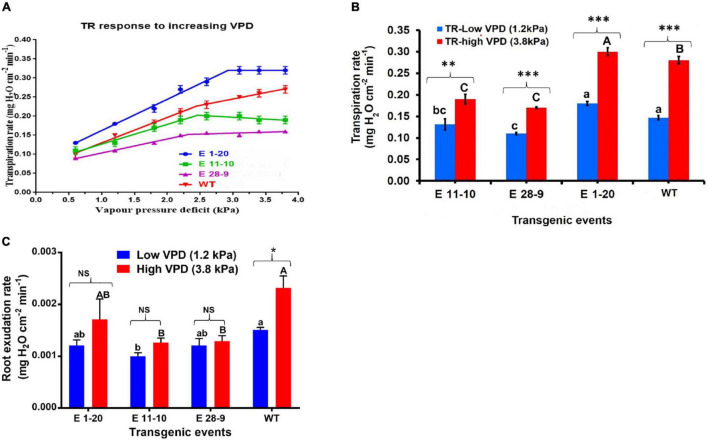
Performance of the transgenic and non-transgenic WT plants under stable and increasing VPD conditions. **(A)** Tr response to increasing VPD conditions in *PgPIP2;6* transgenic and WT tobacco plants. A plot of transpiration rates against VPD for three transgenic tobacco lines [E 1–20 (the blue-color line); E 11–10 (the green-color line); E 28–9 (the pink-color line)] and WT (the red-color line) plants. Compared to WT, transgenic events (except E 1–20) showed limited Tr under high-VPD conditions. Each data point represents means (± SE) six replicates. **(B)** Mean Tr of *PgPIP2;6* transgenic and WT plants under low-VPD and high-VPD conditions. The bars with blue color represent low-VPD (1.2 kPa) Tr, and bars with red color represent high-VPD (3.8 kPa) Tr. Data represent means (± SE) six replicates, and means were analyzed by the Tukey–Kramer test. The “P” indicates the probability of a difference among the transgenic events and WT plants in low and high VPD. Bars with different capital letters and small letters indicate significantly different at *P* ≤ 0.05 in high-VPD and low-VPD conditions. The asterisks indicate the significant difference at *P* ≤ 0.05 between low- and high-VPD treatments. “Bars with asterisk ** and *** symbols are significantly different at *P* < 0.01, *P* < 0.001 between low VPD and high VPD stress treatment. **(C)** The root exudation rate of transgenic and WT tobacco plants subjected to low (1.2 kPa) and high-(3.8 kPa) VPD stress. The exudation rate was calculated by sampling the root sap. Data were analyzed by the Tukey-Kramer test. Values represent means ± SE (*n* = 6). Bars with different capital letters and small letters indicate significantly different at *P* ≤ 0.05 in high VPD and low VPD conditions. Bars with asterisk * symbol is significantly different at *P* < 0.05, between low and high VPD exudation. Bars with NS indicate that both low and high VPD root exudation are statistically non-significant.

Transpiration rate (Tr) was gradually increased under a ladder or a high-VPD (0.6–3.8 kPa) chamber in both transgenic as well as WT plants (Figure 4A). By contrast, Tr was stable or parallel response in a low-VPD (1.2 kPa) chamber in both transgenic and WT plants (data not shown). Under high-VPD conditions, while Tr increased in all the tested genotypes, the slope of Tr decreased beyond the VPD breakpoint (2.4 kPa), thereby indicating limited transpiration response in most of the genotypes (Figure 4A). WT plants displayed higher Tr than the transgenic events (except in the transgenic event E 1–20). Transgenic events, such as E 11–10 and E 28–9, showed lower Tr at high VPD, indicating that they might have water-conserving character. Water conservation mechanisms were highly expressed under high evaporative demand. Mean Tr comparison between transgenic and WT plants in low-VPD and high-VPD-chamber conditions displayed statistically significant genotypic differences at *p* ≤ 0.05 (Figure 4B and Table 2). The root exudation rate was slightly higher when exposed to high VPD than in the low-VPD-exposed plants. Under low- and high-VPD (1.2 and 3.8 kPa) conditions, transgenic plants exhibited slightly lower root exudation rates than WT plants (Figure 4C), thereby implying lower Tr.

**TABLE 2:** Summary of different physiological traits that contribute to different abiotic stress tolerance in PgPIP2;6 transgenic and WT tobacco plants.

S.no	Trait name	E 1–20	E 11–10	E 28–9	WT	L.S.D. (0.05)	*P*-value significance	Stress
1	Transpiration rate – Low VPD (mg H_2_O cm^–2^ min^–1^)	0.18a	0.13bc	0.11c	0.15b	0.019	***	VPD
2	Transpiration rate – High VPD (mg H_2_O cm^–2^ min^–1^)	0.32a	0.19c	0.16c	0.27b	0.024	***	
3	Root exudation rate – Low VPD (mg H_2_O cm^–2^ min^–1^)	0.00121ab	0.001006b	0.00121ab	0.00151a	0.00028	*	
4	Root exudation rate – High VPD (mg H_2_O cm^–2^ min^–1^)	0.001715ab	0.001268b	0.00129b	0.00232a	0.00073	*	
5	Leaf area VPD (cm^–2^)	501c	843b	1091a	1086a	83.709	**	
6	Total chlorophyll content (μmol per m^2^ of leaf)	27.26b	28.03ab	30.33a	25.90b	2.060	*	
7	Total Biomass (g)	3.31c	7.07b	9.46a	6.0415b	1.189	**	
8	Total transpiration-Well watered (g)	1177.11b	1238ab	1217.88b	1370.44a	103.72	**	Progressive drought stress
9	Total transpiration-Well stress (g)	309.41ab	309.5ab	297.58b	325.16a	13.68	**	
10	Total biomass-Well watered (g)	8.8216c	13.6266ab	15.7433a	11.7933a	2.147	**	
11	Total biomass-Water stress (g)	5.39c	9.8244ab	11.333a	8.4133b	1.822	**	
12	Relative water content (%)-Well watered	95.63bc	94.30c	96.39b	98.96a	1.479	***	
13	Relative water content (%)-Water stress	89.40b	93.39a	91.90ab	93.24a	2.045	**	
14	Transpiration- Control at 28°C (g H_2_O in 3 h)	18.46d	28.46b	22.2c	34.26a	2.77	***	Heat
15	Transpiration-Heat stress at 40°C (g H_2_O in 3 h)	46b	56.33ab	62.33ab	74a	19.101	ns	
16	Canopy temperature-Control at 28°C	29.402b	29.163b	28.500c	31.157a	0.307	***	
17	Canopy temperature-Heat stress at 40°C	35.218c	36.230b	36.560b	38.090a	0.482	***	
18	Canopy temperature depression (CTD)-Control at 28°C	-1.403b	-1.163b	-0.500a	-3.156c	0.309	***	
19	Canopy temperature depression (CTD)-Heat stress at 40°C	4.781a	3.769b	3.439b	1.909c	0.483	***	
20	Transpiration- Control at 28°C (g H_2_O in 3 h)	18.46d	28.46b	22.2c	34.26a	1.28	***	Cold
21	Transpiration-Cold stress at 4°C (g H_2_O in 3 h)	15a	15a	16a	21a	4.416	*	

Means with different letters of alphabets are significantly different (P < 0.05) and the same letters represent the absence of significant difference. The asterisk *, **, and *** symbols are significantly different at P < 0.05, P < 0.01, P < 0.001, and ns: non-significant, respectively.

#### Progressive Drought Stress

**FIGURE 5:**
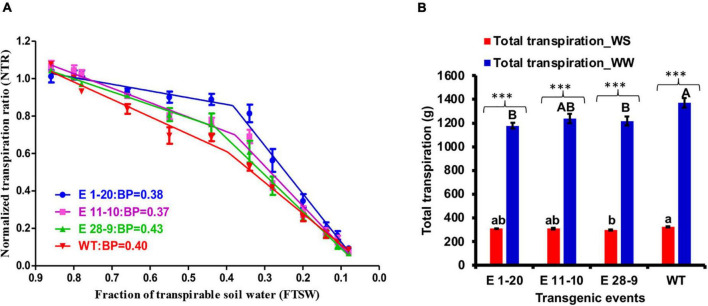
Progressive drought stress. **(A)** Relationship between the normalized transpiration rate (NTR) and fraction of transpirable soil water (FTSW) of transgenic [E 1–20 (blue color line); E 11–10 (pink color line); E 28–9 (green color line)] and WT (red color line) during progressive drought stress treatment. The FTSW thresholds where the transpiration initiated its decline were calculated with segmental regression procedure from Graphpad prism. Then, the regression lines of the relationships between the NTR and FTSW were drawn by fitting NTR to FTSW data above and below the respective thresholds for transpiration decline in each genotype. The dotted lines represent the NTR of well-watered control plants. Each point is the mean of NTR (*n* = 9), and the error bars are the SE of the mean. **(B)** Total transpiration content in *PgPIP2;6* transgenic tobacco lines under well-watered (WW) and water stress (WS) treatment. Bars with different capital letters and small letters indicate significantly different at *P* ≤ 0.05 in both low and high VPD conditions. Bars with asterisk *** symbol are significantly different at *P* < 0.001 between WW and WS treatment.

Imposition of progressive drought stress on transgenic and WT tobacco plants revealed that WT plants utilized soil moisture more quickly than the transgenics that also showed genotypic differences with declined transpiration between WT and transgenic plants (Figure 5A). Segmental regression analysis revealed a slightly higher FTSW breakpoint value in WT plants than the transgenic events (except the event E 28–9), although the events E 1–20 and E 11–10 exhibited a slower decline in transpiration than the WT plants (Figure 5A). Total transpiration was measured in WW as well as in WS plants where a threefold total transpiration difference was recorded (Figure 5B). While under WW conditions, events E 1–20 and E 28–9 were significantly different from WT (Figure 5B and Table 2). Similarly, event E 28–9 was significantly different from WT under WS conditions (Figure 5B and Table 2).

#### Temperature Stress

**FIGURE 6:**
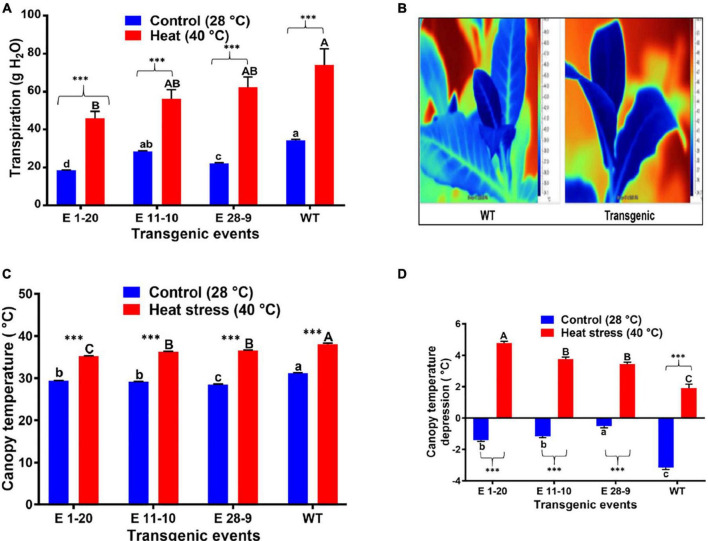
Physiological performance of *PgPIP2;6* transgenic tobacco lines under heat stress at 40°C. **(A)** Tr in transgenic lines and WT plants subjected to heat stress treatment (40°C for 4 h). **(B)** Thermal infrared images of leaf from WT and transgenic tobacco plants. The images were taken after 4 h of heat stress treatment. The color scale to the right shows the variation in leaf temperature produced by heat. **(C)** Canopy temperature of the transgenic lines E 1–20, E 11–10, and E 28–9 and WT tobacco plants under 40°C heat stress and control (28°C) conditions. Measurements were taken after 4 h of stress treatment on three replicates of each line. Data are means ± SE. **(D)** Canopy temperature depression in transgenic tobacco lines and WT plants under heat stress. Data are means ± SE. Bars with different capital letters and small letters indicate significantly different at *P*≤0.05 in both control and heat stress conditions. Bars with asterisk *** symbols from **(A,C,D)** are significantly different at *P* < 0.001, between control and heat stress treatment.

**FIGURE 7:**
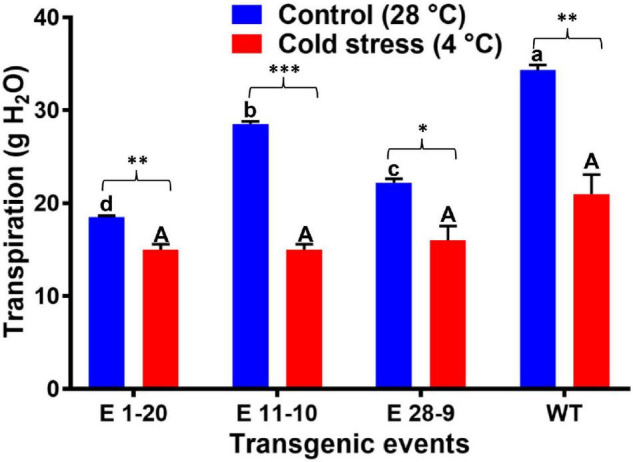
The transpiration rate in transgenic and WT plants subjected to cold stress treatment (4°C for 4 h). Bars with different capital letters and small letters indicate significantly different at *P*≤0.05 in both control and cold stress conditions. Bars with asterisk *, **, and *** symbols are significantly different at *P* < 0.05, *P* < 0.01, *P* < 0.001 between control and cold stress treatment.

Transpiration was measured in both control and heat-stressed plants where significant differences were noticed in their transpiration rates (Figure 6A), transgenics exhibiting lower transpiration than the WT plants (Figure 6A). Thermal image analysis revealed lower canopy temperature (CT) in transgenics than WT plants under high-temperature (40°C) stress (Figure 6B). Similarly, transgenics showed lower CT than the WT plants under optimum (28°C) and high-temperature (40°C) conditions (Figure 6C). Canopy temperature depression (CTD) analysis demonstrated higher CTD in transgenics than WT plants under high-temperature stress conditions (Figure 6D), which reverses the CT measurements. A comparison of the mean CT, CTD, and transpiration data among the transgenic and WT plants in both heat-stressed and control plants (optimum temperature, 28°C) revealed statistically significant (*p* ≤ 0.05) genotypic differences (Table 2). Under cold stress (4°C), there was no significant difference between transgenic and WT plants. By contrast, transgenic events E 1–20, E 11–10, and E 28–9 significantly lower transpiration than WT plants (Figure 7 and Table 2).

### Transgenic Tobacco Plants Have Higher Expression of the *PgPIP2;6* Gene Under Abiotic Stresses

**FIGURE 8:**
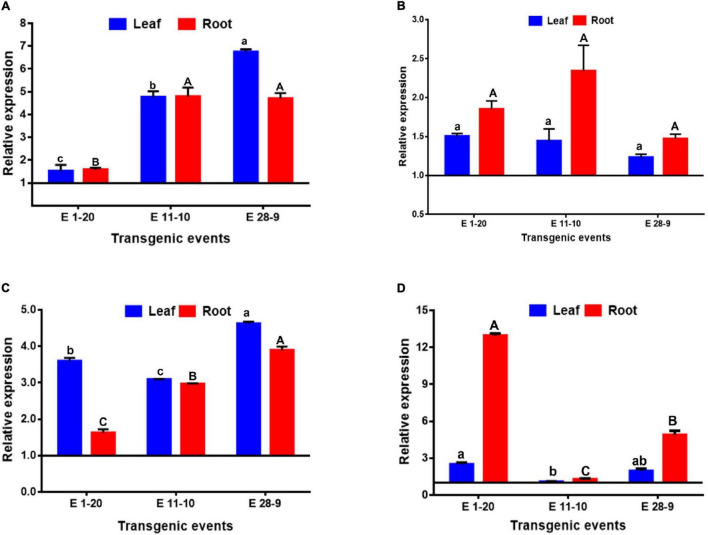
The relative expression of *PgPIP2;6* gene in transgenic tobacco plants under high VPD **(A)**, progressive drought **(B)**, heat **(C)**, and cold **(D)** stresses. *Y*-axis indicates the relative gene expression values, and names on the *X*-axis represent the transgenic events. Dark black-color bars indicate the leaf expression, and gray-color bars indicate root expression. Data points represent the *PgPIP2;6* transgene expression values, obtained after normalization against the reference gene (UBC) and corresponding control samples by 2^–ΔΔCt^ method. Each data point represents mean of three biological replications with standard error (±SE). Each biological replication represents mean of three technical replications. The “P” indicates the probability of a difference among the transgenic events expression in leaf and root tissues. Bars with different capital letters and small letters are indicated significantly different at *P* ≤ 0.05 in root and leaf tissues.

Expression levels of the *PgPIP2;6* gene in leaf and root tissues, subjected to different abiotic stress treatments, were compared with their corresponding control samples using the 2^–ΔΔCt^ method where a steady upregulation was recorded in response to high VPD stress. Expression of *PgPIP2;6* gene increased significantly in all the tested transgenic events (Figure 8A). Its expression in leaves was higher than that in root tissues in all the three transgenic events except for E 1–20 in which the expression levels were comparatively less in both the tissues. Overall, the transgenic event E 28–9 showed the highest expression levels in both leaf and root tissues in comparison with other transgenic events (Figure 8A). These expression results were closely related to Tr data where the event E 1–20 displayed higher Tr than the other event under high-VPD conditions (Figures 4A, 8A). The relative expression of *PgPIP2;6* was investigated under drought *vis-a-vis* WW conditions for each transgenic event. Under WS conditions, significant variation in the abundance of the *PgPIP2;6* transcript was observed among diverse transgenic events (Figure 8B). *PgPIP2;6* transgene expression was upregulated under WS in root as well as in the leaf tissues. Overall, the transgenic event E 11–10 showed significantly higher transcript abundance in root tissues than other transgenic events (Figure 8B). A correlation was noticed between physiological characteristics, the level of expression of *PgPIP2;6*, and drought tolerance. Under heat stress, the *PgPIP2;6* transgene showed significant upregulation in all the transgenic events. Higher expression levels were observed in the leaf compared to root tissues in all transgenics (Figure 8C). Among all the tested transgenic events, E 28–9 showed higher expression in leaf as well as root tissues. These expression results agree with the physiological data (Figure 6) where the events with lower *PgPIP2;6* gene expression showed a higher transpiration rate under high-temperature conditions. Under cold stress, root tissues exhibited comparatively higher gene expression than the leaf where the transgenic event E 1–20 showed the highest expression in root tissues than the other two events (Figure 8D). *PgPIP2;6* expression levels were upregulated in all the transgenic events under cold stress conditions that are similar to the Tr data obtained from the physiological experiments (Figure 7).

## Discussion

Maintaining water budget levels under different abiotic stress conditions is crucial for the plants to survive and to employ several adaptive mechanisms to cope with the environmental stresses (Mahdieh et al., 2008). *AQP* genes play critical roles in water transport, homeostasis, growth, and development of plants, including other organisms (Luu and Maurel, 2005; Galmés et al., 2007; Li et al., 2015; Afzal et al., 2016; Ranganathan et al., 2017). Functional characterization of AQPs in different crop species showed improved tolerance against abiotic stress conditions (Ayadi et al., 2011; Zhou et al., 2012; Liu et al., 2013; Ranganathan et al., 2016; Xu et al., 2020, 2021; Sun et al., 2021). An adaptive mechanism of pearl millet to most of the abiotic stresses also involves this important class of proteins through the regulation of *AQP* genes. The *AQP* gene family has been characterized in diverse taxa, such as *Oryza sativa*, *Zea mays*, *Triticum aestevium*, *Hordeum vulgare*, *Sorghum bicolor*, *Panicum virgatum*, and *Brachypodium distachyon* (Johanson et al., 2001; Forrest and Bhave, 2008; Nguyen et al., 2013; Hove et al., 2015; Azad et al., 2016). However, systematic characterization and functional validation of the *AQP* genes have not been demonstrated in *Pennisetum glaucum*. The present study carried out expression profiles of *AQP* genes in leaf and root tissues of contrasting genotypes under high VPD and progressive drought stresses with the primary objective of elucidating the essential roles being played by AQPs. In all, 34 *AQP* genes were identified in the *P. glaucum* genome that are closer to those from other monocots like rice, *S. bicolor*, and *Z. mays*. Pearl millet AQP proteins contained similar structural motifs like MIP domains, NPA, LE1, LE2, H1, H2, and P1-P5, thereby indicating similar functional roles in pearl millet also. The presence of *cis*-elements in the promoter regions infer that they might play a vital role in developmental and abiotic stress regulations. *PgAQP* genes displayed different expression patterns in different tissues (leaf and root) of the VPD-sensitive and insensitive genotypes, indicating their tissue specific roles during abiotic stress tolerance. Based on the expression data of all *PgAQPs* under high VPD and drought stress to VPD-sensitive and insensitive genotypes, the *PgPIP2;6* gene of the PIP family could be a probable candidate gene that plays a pivotal role during abiotic stress tolerance, particularly drought. This result has been well aligned with our earlier study in pearl millet (Reddy et al., 2017). *PgPIP1;3*, *PgPIP1;4*, and *PgPIP2;3* showed higher transcript abundance in high TR genotype than low TR genotype under high VPD conditions (Reddy et al., 2017; Grondin et al., 2020).

The PIP proteins belong to the AQP family, which are known to play critical roles during water transport in plasma membranes of many plant species (Martre et al., 2002; Tyerman et al., 2002; Song et al., 2016; Zargar et al., 2017) toward abiotic stress adaptation. In our pursuit to identify a functional role of the pearl millet *PgPIP2;6 AQP* gene in transgenic tobacco plants, sequence analysis of the protein with AQPs isolated from other monocot and dicot species revealed that PgPIP2;6 protein shares high sequence homology with ZmPIP2;6 and SbPIP from maize and sorghum where the predicted structure resembles that of a classic AQP. Homology-based modeling revealed the orientation of NPA motifs similar to that of other characterized AQPs, besides the capacity to form homotetramers that are typical of AQPs. The *PgPIP2;6* gene sequence is similar to other well-characterized AQP sequences with all the essential components for its function. Transgenic lines with the *PgPIP2;6* gene under the control of the constitutive *CaMV35S* promoter thus generated were advanced to T_2_ generation where three events (E 1–20, E 11–10, and E 28–9) were selected based on initial molecular characterization for elucidation of the function of the *PgPIP2;6* gene under different abiotic stress conditions.

In the present study, *PgPIP2;6* transgenic tobacco plants exhibited lower Tr at high VPD than WT plants, indicating that the transgene enabled the plants with water-conserving properties. Genotypic differences for the transpiration response to increasing VPD have also been reported in other crop species like soybean (Fletcher et al., 2007; Gilbert et al., 2011), sorghum (Gholipoor et al., 2010), peanut (Devi et al., 2010), pearl millet (Kholová et al., 2010b; Reddy et al., 2017, 2021), cowpea (Belko et al., 2012), maize (Yang et al., 2012; Gholipoor et al., 2013), and chickpea (Zaman-Allah et al., 2011a; Sivasakthi et al., 2017b). The Tr response of transgenic chickpea plants was shown to be associated with lower Tr slopes than WT control plants under increasing VPD conditions (Anbazhagan et al., 2015). The root exudation rate also showed a clear genotypic difference between WT and transgenics where the transgenic plants showed a lower exudation rate than the WT plants. Such a water conservation strategy has been shown to be directly linked to increased crop production under water-limited conditions (Zaman-Allah et al., 2011b; Vadez et al., 2013, 2014; Kholová et al., 2014). These results are in accordance with the previous reports in pearl millet, sorghum, and chickpea (Choudhary et al., 2013; Sivasakthi et al., 2017a,b; Tharanya et al., 2017). *PgPIP2;6* transcript levels in transgenic tobacco were closely related with the Tr data where transgenic lines displayed lower Tr under high-VPD conditions. Exposure of plants to high VPD is known to reduce the stomatal apertures to control water losses (Kholová and Vadez, 2013). Since AQPs play a critical role in stomatal movements and regulation (Li et al., 2004), tobacco transgenics overexpressing *PgPIP2;6* exhibited lower Tr in high-VPD conditions when compared to the WT controls. This suggests that these plants may have better water-conserving mechanisms by enhancing their transpiration efficiency, thereby leading to better adaptation to water-limiting conditions.

Controlled progressive drought stress imposition is an important methodology for assessing crop performance under water-limiting environments (Kholová et al., 2010a) where the FTSW is an important parameter for drought adaptation. In the present study, progressive drought stress imposed on transgenic tobacco plants resulted in a lower FTSW threshold value than the WT plants, resulting in a slower decline of soil moisture in transgenic plants, possibly due to their water-saving characteristics as also shown in chickpea (Anbazhagan et al., 2015). Genotypic differences were also found for transpiration response to progressive water-deficit stress in several other crops (Vadez and Sinclair, 2001; Bhatnagar-Mathur et al., 2007; Hufstetler et al., 2007; Devi et al., 2010; Anbazhagan et al., 2015). A correlation between physiological characteristics and expression of *PgPIP2;6* transgene seems to be responsible for drought tolerance in our study where the physiological mechanisms linked to drought stress adaptation/tolerance are closely related to the expression of drought-responsive genes (Kiani et al., 2007). Expression of the *PgPIP2;6* gene in root and leaf tissues under water-deficit conditions further supports the role in stress tolerance. Water transport through roots depends on the anatomical structure of roots regulated by the complex phenomenon. Plants growing under adverse conditions, including drought, reduce the water permeability of their root membrane cells to avoid possible loss of water from the roots to the soil, as has been proposed earlier (Steudle, 2000). Hence, the lower Tr found in *PgPIP2;6* transgenic tobacco plants compared to WT plants presumably developed the adaptative mechanism. These results are consistent with the earlier reports on chickpea and pearl millet (Sivasakthi et al., 2017b; Tharanya et al., 2018).

Heat stress lowers the relative air humidity if water is supplemented to the ambient atmosphere (Prasch and Sonnewald, 2013), a scenario frequently occurring under natural climatic conditions and exerted in regular heat-stress studies. Consequently, plants suffer from heat stress and additional ambient water stress (Georgii et al., 2017). High temperature enhances transpiration to minimize heat damage by transpiration-mediated leaf cooling (Crawford et al., 2012). For any given environmental conditions, the canopy temperature (CT) is closely related to the rate of transpiration from the canopy surface, while the canopy temperature depression (CTD) indicates the capacity of stomata to regulate leaf water loss under stressed conditions (Isoda and Wang, 2002). Therefore, the differences in CT could possibly be used to estimate the differences in Tr, providing a more relevant trait proxy for Tr (Jones et al., 2002). Our results of thermal image analysis revealed that transgenic plants displayed lower CT than WT plants under high-temperature (40°C) conditions and optimum temperatures (28°C). Transgenics exhibited more CTD than WT plants under high-temperature stress conditions, inferring the adaptation of a possible water-saving strategy. During heat stress, plant-water relations are regulated through changes in abundance of AQPs (Maurel et al., 2008). *AQP* genes are expressed differentially in diverse tissues under various abiotic stress conditions in higher plants. Accordingly, it is noticed that *PgPIP2;6* gene expression levels were significantly upregulated under high-temperature conditions in transgenic tobacco lines. This leads us to speculate that the upregulation of *PgPIP2;6* might be a spontaneous response to heat stress and might play a critical role in speeding up the water homeostasis, following the stress.

Aquaporins have also been shown to respond to various environmental stresses, including cold (Jang et al., 2004; Aroca et al., 2005; Guo et al., 2006; Peng et al., 2008; Sade et al., 2010) that may be directly related to their function in the transport of water across membranes. In the present study, *PgPIP2;6* transgenic lines of tobacco displayed slightly lower transpiration than the WT control plants under cold stress conditions. *PgPIP2;6* expression levels were significantly upregulated in transgenics under cold stress except in the line E 11–10, which displayed comparatively lower expression and higher transpiration than the other two transgenics. Similarly, overexpression of *OsPIP2;7* has been shown to enhance the Tr, leading to improved tolerance against low temperature in rice (Li et al., 2008). Leaf dehydration is caused by the imbalance between water lost by leaf transpiration and water taken up by the roots (Pardossi et al., 1992; Vernieri et al., 2001), resulting in a decrease in VPD between the atmosphere and the leaf surface, and leading to a decline in the transpiration rate (Aroca et al., 2003). Hence, under low-temperature stress, stomata of the tolerant transgenic plants close more rapidly (Aroca et al., 2001, 2003; Bloom et al., 2004). Besides, the recovery of plants from cold stress has been linked with the expression of AQPs, particularly PIPs (Afzal et al., 2016).

In conclusion, our study identified 34 *AQP* genes in pearl millet, which are grouped into four subfamilies, including 11 PIPs, 9 TIPs, 11 NIPs, and 3 SIPs. Phylogenetic relationship within the subfamilies of PgAQPs and between AQPs of related species shows their evolutionary relationship and possible functions with their localizations, mainly in the plasma membrane. *Cis*-motif analysis showed the presence of several tissue and abiotic stress-specific *cis-*elements, indicating their possible role during abiotic stress adaptation. Expression analysis of *PgAQPs* in Tr-contrasting genotypes of pearl millet in leaf and root tissues in VPD and progressive drought stress revealed their critical roles during abiotic stress conditions. Tobacco transgenics constitutively expressing *PgPIP2;6* gene showed differences in response to diverse abiotic stresses. Tr under heat and drought stresses recorded marked differences between transgenics and the non-transgenic WT plants, where the transgenics exhibited better tolerance due to lower Tr when compared to WT plants. Constitutive expression of pearl millet aquaporin *PgPIP2;6* in tobacco seemed to impart abiotic stress tolerance with reduced transpiration, thereby helping in water conservation. Further improvement in stress tolerance is possible by using tissue-specific or stress-inducible promoters for more specific *AQP* expression levels. Thus, these studies provide a point of reference for the functional studies and molecular breeding of a pearl millet crop for enhanced adaptation and resilience to abiotic stresses, especially under uncertainties due to the ensuing climate change.

## Data Availability Statement

The datasets presented in this study can be found in online repositories. The names of the repository/repositories and accession number(s) can be found in the article/Supplementary Material.

## Author Contributions

PR, PB-M, VV, and KKS conceived the idea and designed the project outline. PR, MD, KS, KD, KSC, and PK performed the experiments. PR, VV, PB-M, KS, PK, MN, and KKS analyzed the data. PR, MD, KS, KD, KKS, and PK prepared and refined the manuscript. All authors read and approved the manuscript.

## Conflict of Interest

The authors declare that the research was conducted in the absence of any commercial or financial relationships that could be construed as a potential conflict of interest.

## Publisher’s Note

All claims expressed in this article are solely those of the authors and do not necessarily represent those of their affiliated organizations, or those of the publisher, the editors and the reviewers. Any product that may be evaluated in this article, or claim that may be made by its manufacturer, is not guaranteed or endorsed by the publisher.

## Footnotes

1
http://www.gigadb.org/dataset/100192

2
http://gsds.cbi.pku.edu.cn

3
http://www.genome.jp/tools/motif/

4
http://web.expasy.org/protparam

5
http://www.genscript.com/wolf-psort.html

6
http://www.cbs.dtu.dk/services/TMHMM/

7
http://www.cbs.dtu.dk/services/NetPhos/

8
http://meme-suite.org/tools/meme

9
http://bioinformatics.psb.ugent.be/webtools/plantcare/html/

10
https://swissmodel.expasy.org

11
https://bioinfo3d.cs.tau.ac.il/PatchDock

12
http://bioinfo3d.cs.tau.ac.il/FireDock

13
https://meme-suite.org/meme/tools/meme

## Supplementary Material

Supplementary Figure 1 Phylogenetic relationship of *Pg* Aquaporin proteins with *Zea mays* (Zm), *Sorghum bicolor* (Sb), and *Oryza sativa* (Os). Numbers on branches represent bootstrap values. Aquaporins in plants are broadly categorized into four subfamilies, PIPs (plasma membrane intrinsic proteins), TIPs (tonoplast intrinsic proteins), NIPs (nodulin-26-like intrinsic proteins), and SIPs (small basic intrinsic proteins).

Supplementary Figure 2 Conserved protein motifs distribution in 34 PgAQPs. Conserved motifs were analyzed by the MEME Web server using their respective protein sequences. Ten conserved motifs were identified, and different motifs are assigned different colors. The scale represents the lengths of the proteins and motifs.

Supplementary Figure 3 MEME identified conserved motif sequences in PgAQPs.

Supplementary Figure 4 PgAQPs amino acid sequences were aligned using MacVector software. The red line on top of the sequences indicates predicted the MIP domain, and the blue-color portion of the alignment indicates predicted transmembrane domains. The NPA motifs, tetrad residues of ar/R filter, and FPs are highlighted. The two conserved NPA motifs are bold and highlighted in pink color. The residues at H2, H5, LE1, and LE2 of the ar/R filter and forger’s positions (P1–P5) are marked in yellow and green letters, respectively. Conserved residues are represented.

Supplementary Figure 5 Expression analysis of the *PgPIP2;6* gene in pearl millet by qRT-PCR. The relative expression level of *PgPIP2;6* in different tissues **(A)** and under different abiotic stress conditions **(B)** (cold, 4°C; heat, 45°C; drought and salt, 250 mM; and ABA, 100 μM). The data represent the mean ± SE of three replicate samples.

Supplementary Table 1 Primers used in the gene expression analysis.

Supplementary Table 2 Types of protein kinases and phosphorylation sites in the phosphorylation events of PgAQPs.

Supplementary Table 3 The paralogous duplication events and non-synonymous to synonymous substitution ratios (dN/dS) of AQPs.

Supplementary Table 4 The orthologs and non-synonymous to synonymous substitutions of AQPs orthologs of *Pennisetum glaucum*, *Sorghum bicolor*, *Oryzasativa*, and *Zea mays.*

Supplementary Table 5 A vapor pressure deficit (VPD) ladder set-up in a controlled-growth chamber.
