# Supplementary material for: Pearl Millet Aquaporin Gene PgPIP2;6 Improves Abiotic Stress Tolerance in Transgenic Tobacco
Source: Front Plant Sci. 2022 Mar 9;13:820996. doi: 10.3389/fpls.2022.820996 (PMC8959815; doi:10.3389/fpls.2022.820996)
Supplement: Supplementary Figure 1 — Phylogenetic relationship of Pg Aquaporin proteins with Zea mays (Zm), Sorghum bicolor (Sb), and Oryza sativa (Os). Numbers on branches represent bootstrap values. Aquaporins in plants are broadly categorized into four subfamilies, PIPs (plasma membrane intrinsic proteins), TIPs (tonoplast intrinsic proteins), NIPs (nodulin-26-like intrinsic proteins), and SIPs (small basic intrinsic proteins). [file Presentation_1.PPTX]

## Slide 1
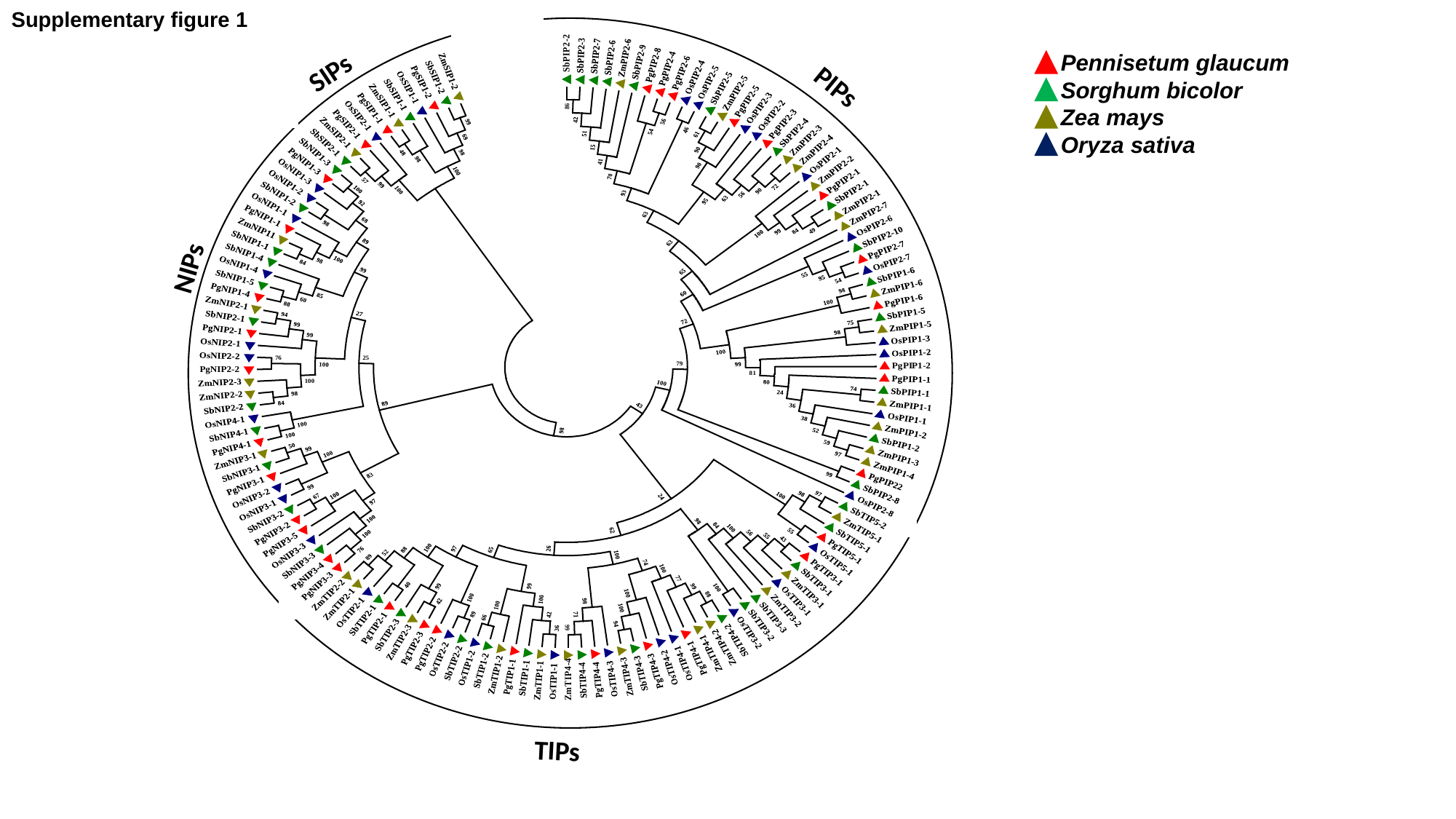

SIPs
PIPs
NIPs
TIPs
Supplementary figure 1
Pennisetum glaucum
Sorghum bicolor
Zea mays
Oryza sativa

## Slide 2
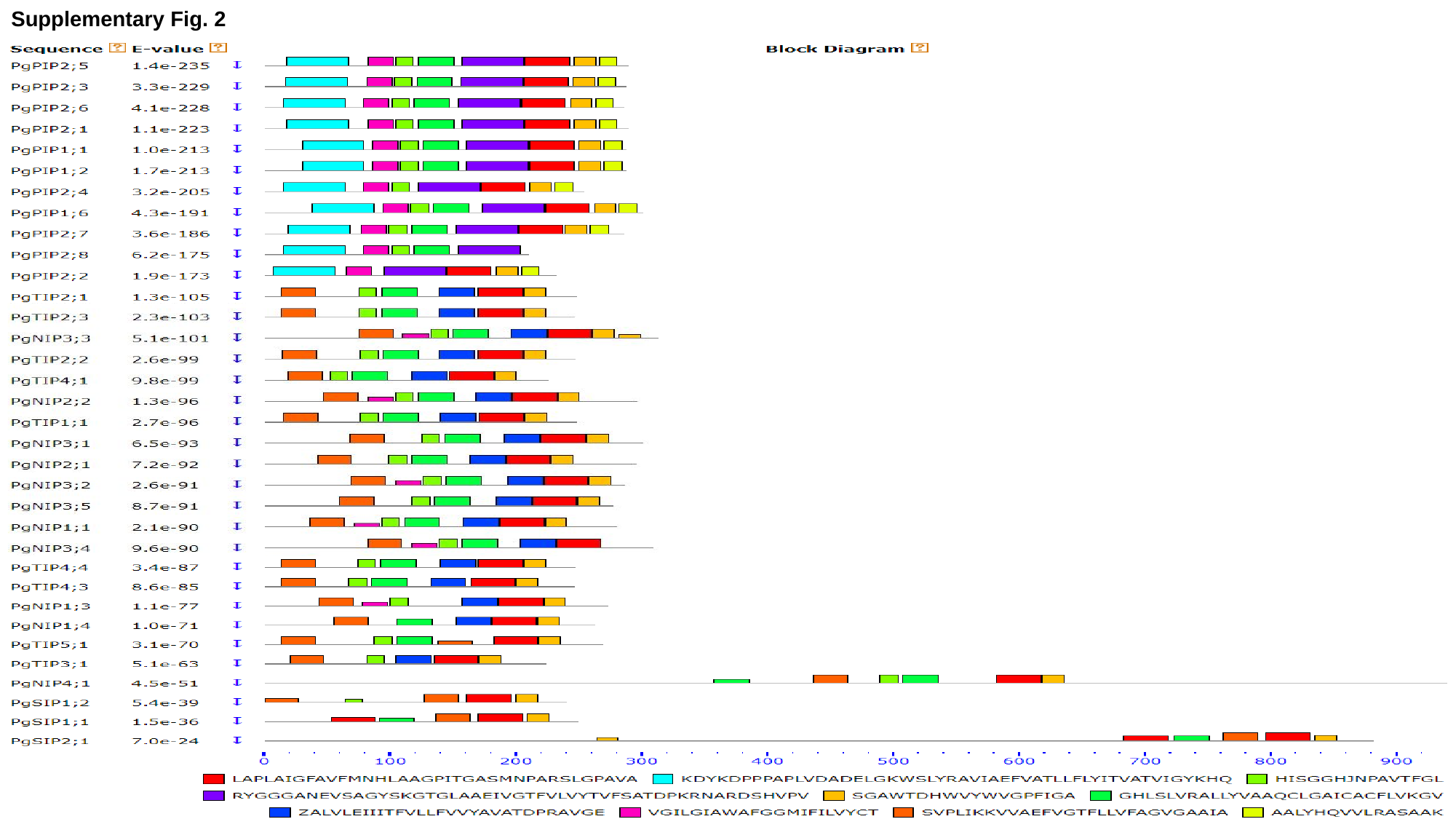

Supplementary Fig. 2

## Slide 3
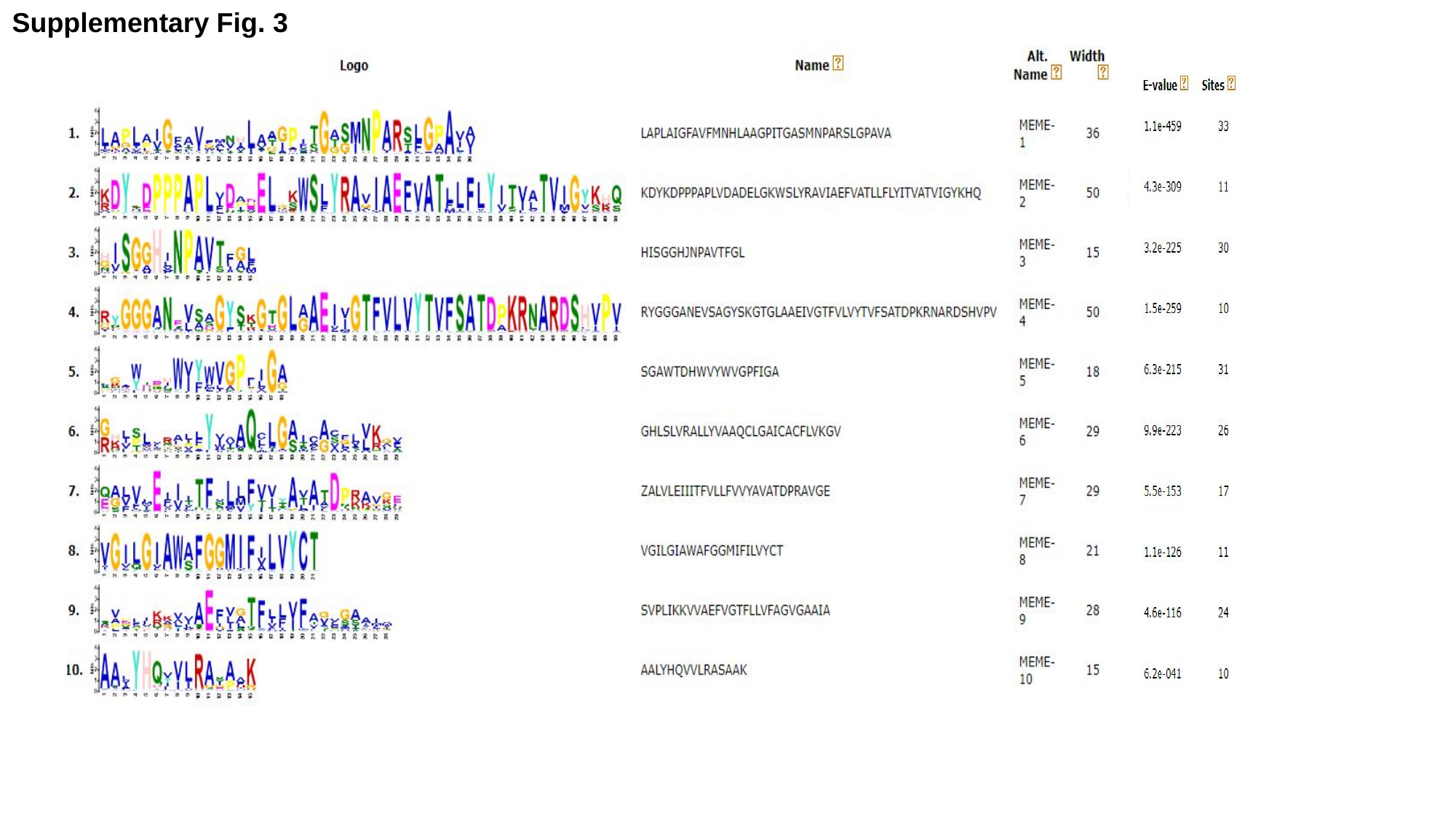

Supplementary Fig. 3

## Slide 4
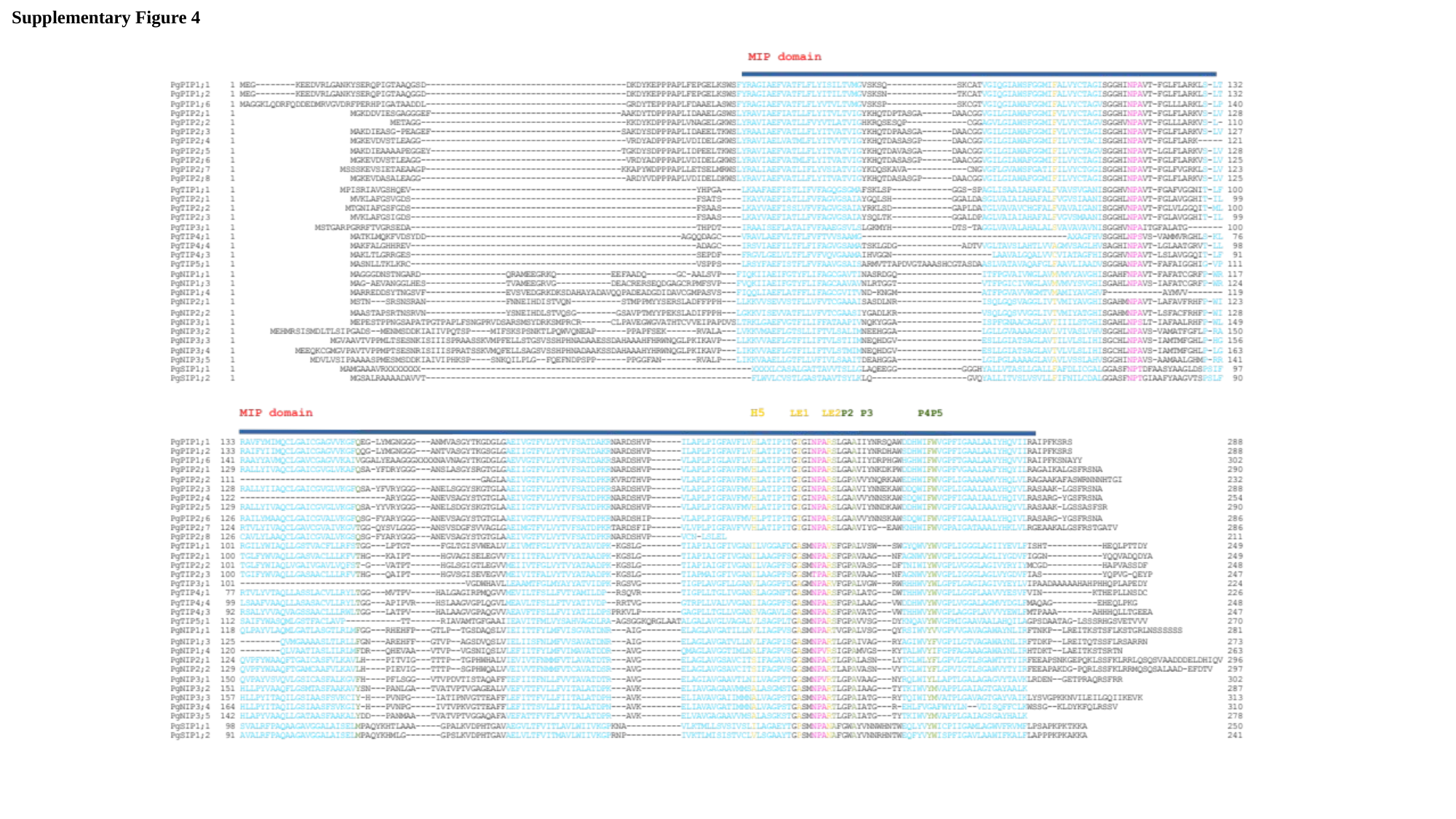

Supplementary Figure 4

## Slide 5
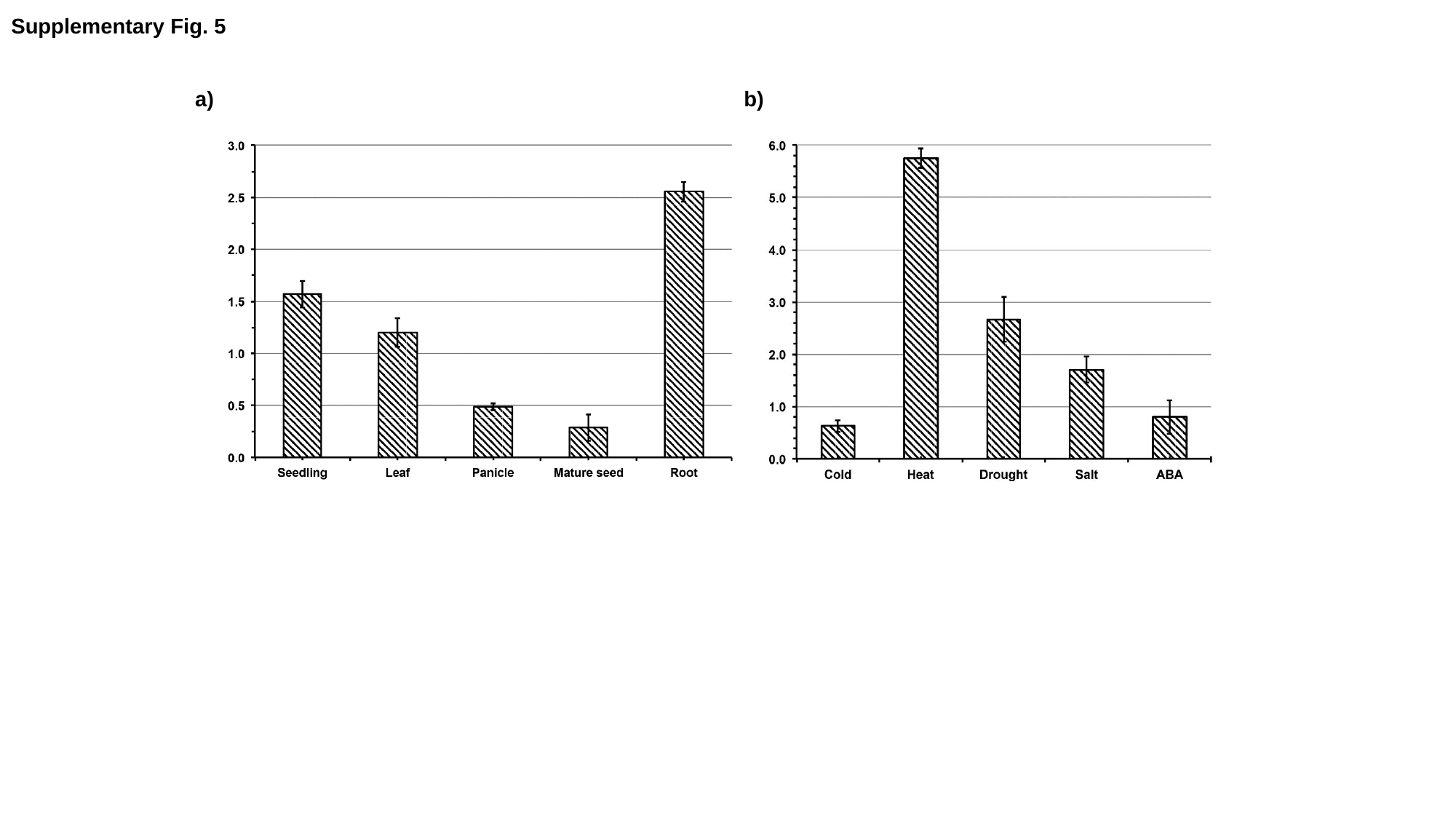

Supplementary Fig. 5
a)
b)
